# Microbial inoculum effects on the rumen epithelial transcriptome and rumen epimural metatranscriptome in calves

**DOI:** 10.1038/s41598-024-65685-y

**Published:** 2024-07-23

**Authors:** P. Fregulia, T. Park, W. Li, L. M. Cersosimo, G. I. Zanton

**Affiliations:** 1 United States Department of Agriculture (USDA) - Agricultural Research Service, Dairy Forage Research Center, Madison, WI USA; 2 Oak Ridge Institute for Science and Education, Oak Ridge, TN USA; 3 Present Address: Tansol Park, Department of Animal Science and Technology, Chung-Ang University, Anseong, South Korea; 4 Present Address: Laura Cersosimo, Brigham and Women’s Hospital, Boston, MA USA

**Keywords:** Early inoculation, Metatranscriptomics, Transcriptomics, Dairy calves, Applied microbiology, Transcriptomics, RNA sequencing

## Abstract

Manipulation of the rumen microbial ecosystem in early life may affect ruminal fermentation and enhance the productive performance of dairy cows. The objective of this experiment was to evaluate the effects of dosing three different types of microbial inoculum on the rumen epithelium tissue (RE) transcriptome and the rumen epimural metatranscriptome (REM) in dairy calves. For this objective, 15 Holstein bull calves were enrolled in the study at birth and assigned to three different intraruminal inoculum treatments dosed orally once weekly from three to six weeks of age. The inoculum treatments were prepared from rumen contents collected from rumen fistulated lactating cows and were either autoclaved (control; ARF), processed by differential centrifugation to create the bacterial-enriched inoculum (BE), or through gravimetric separation to create the protozoal-enriched inoculum (PE). Calves were fed 2.5 L/d pasteurized waste milk 3x/d from 0 to 7 weeks of age and texturized starter until euthanasia at 9 weeks of age, when the RE tissues were collected for transcriptome and microbial metatranscriptome analyses, from four randomly selected calves from each treatment. The different types of inoculum altered the RE transcriptome and REM. Compared to ARF, 9 genes were upregulated in the RE of BE and 92 in PE, whereas between BE and PE there were 13 genes upregulated in BE and 114 in PE. Gene ontology analysis identified enriched GO terms in biological process category between PE and ARF, with no enrichment between BE and ARF. The RE functional signature showed different KEGG pathways related to BE and ARF, and no specific KEGG pathway for PE. We observed a lower alpha diversity index for RE microbiome in ARF (observed genera and Chao1 (p < 0.05)). Five microbial genera showed a significant correlation with the changes in host gene expression: *Roseburia* (25 genes), *Entamoeba* (two genes); *Anaerosinus*, *Lachnospira*, and *Succiniclasticum* were each related to one gene. sPLS-DA analysis showed that RE microbial communities differ among the treatments, although the taxonomic and functional microbial profiles show different distributions. Co-expression Differential Network Analysis indicated that both BE and PE had an impact on the abundance of KEGG modules related to acyl-CoA synthesis, type VI secretion, and methanogenesis, while PE had a significant impact on KEGGs related to ectoine biosynthesis and D-xylose transport. Our study indicated that artificial dosing with different microbial inocula in early life alters not only the RE transcriptome, but also affects the REM and its functions.

## Introduction

Adult ruminants are in a symbiotic relationship with a complex microbial community inhabiting the rumen that converts low quality, human undigestible plant fiber into the substrates for the production of high-quality animal protein^1^. This rumen microbial community is composed of species from all domains of life and includes bacteria, archaea, protozoa, and fungi^2^. The rumen microbiota are divided into three main groups: microorganisms attached to the undigested solid feed fraction residing within the rumen, microorganisms free-floating in the liquid fraction, and microorganisms attached to the rumen epithelium termed epimural microbiota^3,4^. Studies have shown that the taxonomic composition of these three groups are substantially different, with most studies characterizing the rumen microbiota in solid and liquid fractions^5–7^, and fewer studies evaluating the rumen epimural microbiota^8,9^, despite its important and unique role in the interaction between microbial metabolism and the host^10^. Anderson and co-authors^4^, in a meta-analysis, established the core bacterial microbiota of the rumen epithelium (RE) using DNA-based, 16S rRNA amplicon sequencing datasets. However, several aspects of RE microbiota remain unexplored. They include the active microbial communities in the RE in response to diet treatments and manipulation early in life. Additionally, 16S rRNA amplicon sequencing is limited in its ability to detect a full spectrum of RE microbiome due to the pre-defined primer sets used in amplicon capture.

Manipulation of the rumen microbial ecosystem is one potential approach to improve rumen fermentation and consequently enhance the host productive performance^1,11^. Inoculation of exogenous ruminal microorganisms in adult ruminants has resulted in minor effects on rumen fermentation, with microbial populations tending to revert to the original composition after a short period of time even with a near-complete exchange of ruminal contents between animals^12,13^. However, Yañez-Ruiz and co-authors^14^ reported that different diets impacted bacterial colonization during the weaning period in lambs, and this effect persisted over months. Therefore, manipulation of the rumen in early life when the rumen and its microbial community are undeveloped is a potential approach to direct the rumen ecology that will persist into adulthood and contribute to rumen health, production, and efficiency^15^.

Maturation and functionality of the RE tissue generally occurs simultaneously with the gradual acquisition of microorganisms^16,17^. The RE is a unique place of interaction between microbial community and its host, promoting the exchange of the end products from the rumen metabolism (e.g. volatile fatty acids) between the rumen environment and the bloodstream^18^. The rumen epithelium is stratified, while the presence of papillae increases the absorptive surface area and allows increased microbial attachment to the rumen wall^19^. The RE tissue has an intensive metabolic activity and plays a fundamental role in the absorption and metabolism of volatile fatty acids (VFA) produced by microbial fermentation along with immune and barrier functions^20–22^.

We previously reported the results of two types of adult-derived rumen inoculum (bacterial-enriched [BE] and protozoal-enriched [PE] inoculum), which were dosed in pre-weaned dairy calves and their effect was evaluated in pre- and post-weaning periods^23,24^. We found that inoculation resulted in minor changes in microbial abundances in the rumen fluid and in the calf health and growth. However, microbial network analysis showed specific co-occurrence and mutually exclusive interactions related to each treatment^23,24^. Due to the importance of the RE tissue and epimural microbial community to dairy calf health and nutrition, the objective of this study was to evaluate the influence of inoculating pre-weaned dairy calves with three different types of microbial inoculum on the REM and on RE transcriptome. We hypothesized that inoculation of young calves with different types of adult dairy cow rumen fluid could modify REM and the expression of RE transcriptome profiles.

## Results

### RNA reads and quality for rumen epithelium tissue

The extracted RNA samples from RE tissue were of high quality, with an average RNA integrity number of 8.3 ± 0.35. An average of 33.3 M ± 0.7 M total number of raw reads were obtained for each sample. The total number of expressed genes (FPKM ≥ 1) ranged from 13,669 to 14,287 across the samples. All samples had a similar distribution of gene expression, with most of the genes expressed in the range of 0.3 to 5 FPKMs. Good’s coverage was higher than 99% in all RNA-seq samples.

### Gene expression profile and related pathway analysis

The RE tissue had a total of 270 differentially expressed genes (DEG) by pairwise comparison between the treatment groups. There were 127 DEGs between the BE and PE groups (adjusted-p < 0.05 and fold-change > 1.5), with 13 up-regulated genes (URGs) in the BE group and 114 URG in the PE group. There were 36 DEGs between ARF and BE, with 27 URGs in ARF and 9 URGs in BE. There were 107 DEGs between ARF and PE, with 15 URGs in ARF and 92 in PE (Supplementary Table S1).

Using the DEGs obtained by pairwise comparison between the treatment groups, we performed the GO analysis and used REVIGO to remove redundant GO terms. We found 21 enriched GO terms between ARF and PE, 33 enriched terms between BE and PE, and no enriched terms were found between the ARF and BE groups. Then, we used the DEGs to perform a biological process analysis of GO. The analysis of the GO terms related to Biological Process (BP) presented the same GO terms than the analysis of all the GO categories (molecular function (MF), cellular component (CC) and biological process (BP), with 21 enriched GO terms between ARF and PE, 33 enriched terms between BE and PE, and no enrichment between ARF and BE (Supplementary Table S1).

Among the top 5% most highly expressed genes for the three treatment groups, 31 genes were uniquely expressed in the ARF group (*PLD1*, *EIF3L*, *MAPK13*, *AMFR*, *FH*, *CHMP5*, *CBR4*, *ABHD11*, *NUTF2*, *SLC31A1*, *ARHGDIA*, *AES*, *EMC10*, *ATF4*, *PET100*, *HNRNPAB*, *TMEM258*, *OCIAD2*, *PSMB7*, *CHCHD3*, *SCGB1C1*, *UBL5*, *TRA2B*, *UTP14A*, *PPP1R14B*, *MYH9, TPRG1L, ERH, CTTN, PLK2,* and *SNRPG*), majority of these genes were enriched in pathways related to cellular component, including intracellular-bounded organelle (GO:0043231, 29 genes, p < 0.0005) and cytoplasm (GO:0005737, 27 genes, p < 0.005). For the BE group, 20 genes were uniquely expressed (*LAMTOR4, SULT1B1, ACO1, PPARG, SLC26A2, ARL6IP1, HECTD1, TMOD3, FXYD3, RBP4, CCNI, FEM1B, STT3B, TRAM1, DDX6, EZR, FUCA1, BZW2, TM9SF3,* and *ACTA2*) (Supplementary Table S1). GO enrichment analysis indicated enrichment in positive regulation of TOR signaling (GO:0032008, 4 genes, p < 0.004) and cellular response to amino acid stimulus (GO:0071230, 4 genes, p < 0.004). For the PE group, 6 genes were uniquely expressed (*MORF4L2, NOV, YWHAG, FBLN1, ME1*, and *BRP44L*). No significant GO terms were identified for these genes. Using the unique genes, we used the Gene Ontology analysis to explore the gene functions. The GO enrichment analysis showed enriched terms on ARF, BE or PE groups (p < 0.05). Using the unique genes for each group, we performed biological process (BP) analysis of GO. The BP analysis of the ARF group indicated enrichment in cellular component organization or biogenesis (GO:0071840, 16 genes, p < 0.03), cellular component organization (GO:0,016,043, 15 genes, p < 0.04), and cellular component biogenesis (GO:0044085, 11 genes, p < 0.04). The BP analysis of the BE group indicated enrichment in localization (GO:0051179, 13 genes, p < 0.006), protein metabolic process (GO:0019538, 11 genes, p < 0.02), and protein localization (GO:0008104, 9 genes, p < 0.002). We found no enriched terms in BP for the PE group (Supplementary Table S1).

### Rumen epimural microbiota community structure

The three types of inoculums resulted in no differences in Evenness, Shannon diversity index, and Simpson’s index. However, the Observed genera and Chao1 estimates differ by inoculations (Table 1), whereas microbial Observed genera was significantly higher in PE inoculum (p < 0.05).

**Table 1:** Microbial diversity measures from calves dosed with three different types of artificial dosing of rumen inoculum.

Diversity measurements	ARF inoculum	BE inoculum	PE inoculum	SE	p-value
Observed genera	991	1208	1403	61.42	0.005
Chao1 estimates	1380	1648	1822	69.08	0.01
Evenness	0.54	0.52	0.59	0.02	0.31
Shannon diversity index	5.42	5.36	6.2	0.22	0.23
Simpson’s index	0.93	0.9	0.96	0.02	0.44

Diversity measures are represented as the mean ± standard error (SE).

### Taxonomic microbial profile and microbial signature by inoculum type

After using SILVA reference to classify the microbial data, taxonomic profiling revealed a total of 125 taxa at the phylum level and 2,341 taxa at the genus level in the rumen epithelium. A complete list of all taxa classified is provided in Supplementary Table S2.

**Figure 1:**
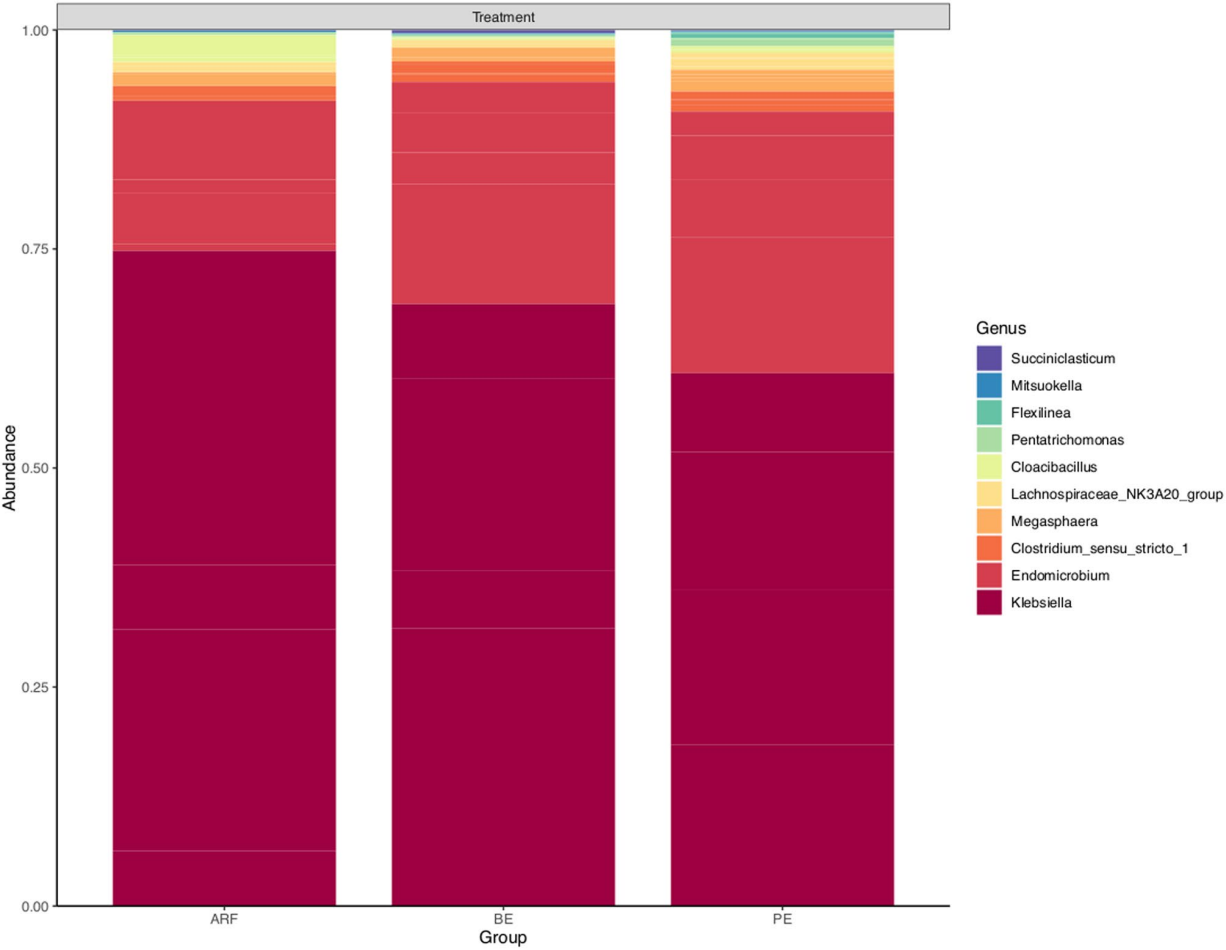
Taxa summary plot for microbial abundance across the ARF, BE, and PE groups. Considering the total abundance, the ten most abundant microbial genera were plotted.

Nine of the ten most abundant genera are prokaryotes, belonging to the genera *Klebsiella* (9%), *Endomicrobium* (3%), *Clostridium* sensu stricto 1 (0.3%), *Megasphaera* (0.2%), *Cloacibacillus* (0.2%), *Lachnospiraceae* NK3A20 group (0.1%), *Flexilinea* (0.02%), *Mitsuokella* (0.02%), and *Succiniclasticum* (0.02%); and one Eukaryotic genus, *Pentatrichomonas* (0.05%) (percentages relative to the total abundance). The ten most abundant taxa were plotted on Fig. 1.

**Figure 2:**
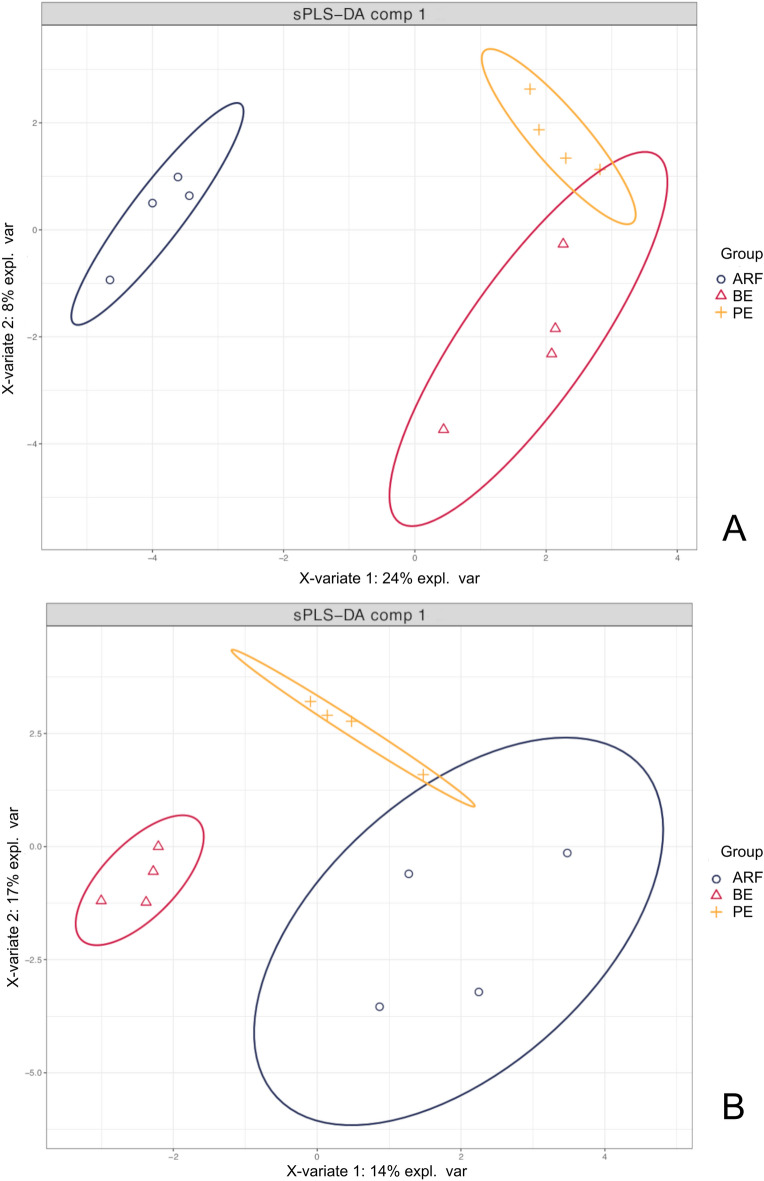
Results of sPLS-DA for microbial the profile at genus level in the microbiome attached to the rumen epithelium of calves that received three types of inoculums. Individual score plot of the samples along the first two components**,** with a 95% confidence level. (**A**) taxonomic profile; (**B**) functional profile.

The sPLS-DA multivariate analysis implemented in the mixOmics R package was used to identify microbial taxa that best characterize each treatment group with 95% of confidence. For this analysis, only microbial taxa with a relative abundance > 0.01% across all the samples were considered. After the centered log-ratio transformation procedures, it was observed a separation in microbial taxonomic profile differentiating the rumen microbiota in ARF, BE and PE groups (Fig. 2A). The BE and PE taxonomic profile overlapped, indicating that the structures of the microbial communities from both groups were partially similar. On the other hand, the functional profile of the BE group showed a clear separation from ARF and PE (Fig. 2B). In contrast to the taxonomic profile, the functional profile showed that ARF and PE groups overlap, while the BE group is clearly different (Fig. 2B).

**Figure 3:**
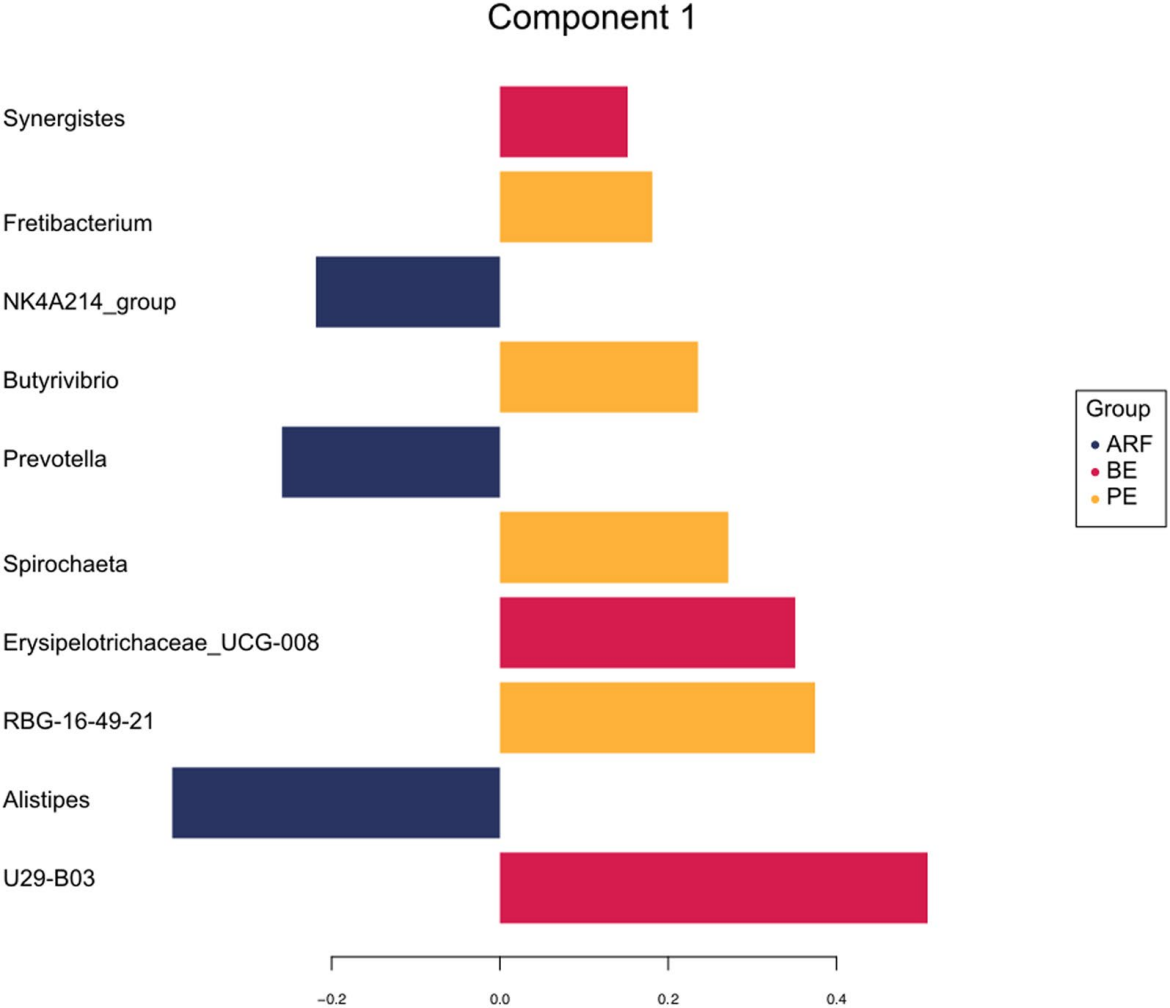
Discrimination of the taxa that best characterize each treatment. The loading plot displays the abundance of the microbial taxa on the treatment they are the most relevant. Ranked from the bottom (most important) to the top. Colors indicate the type of inoculum received in which the microbial taxa was most relevant.

Overall, 40% of the microbial signatures selected in component 1 of the sPLS-DA characterized the rumen microbiota of animals treated with PE inoculum, which included members of the taxa RBG−16−49−21, *Spirochaeta*, *Butyrivibrio*, and *Fretibacterium*. Alternatively, the microbial signature that characterized the BE group represented 30% of the microbial signature selected in component 1, and contain the taxa U29−B03, *Lachnospiraceae* UCG−008, and *Synergistes*. The microbial signature that characterized the ARF group represented 30% of the microbial signature and included the taxa *Alistipes*, *Prevotella*, and NK4A214 group (Fig. 3).

**Figure 4:**
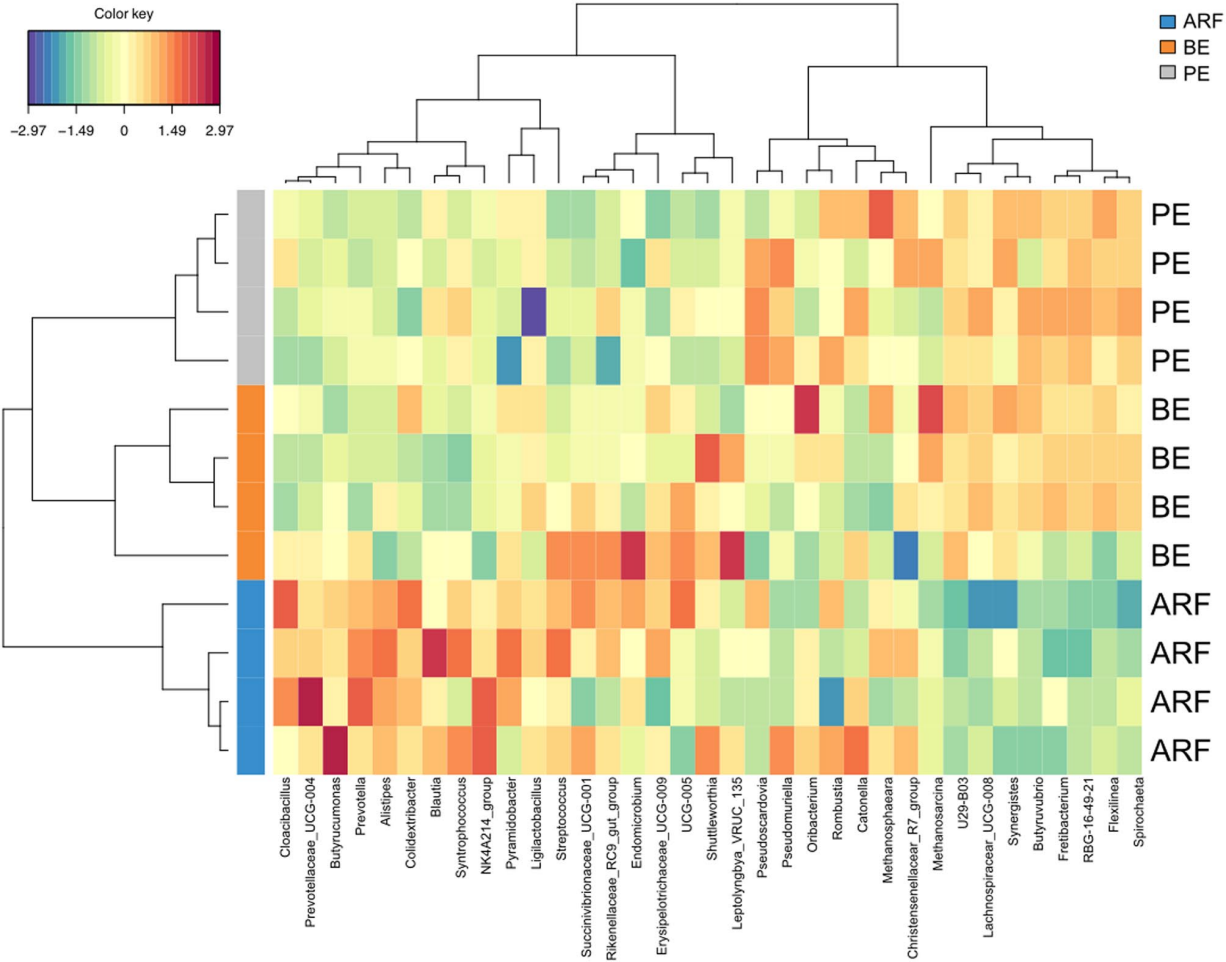
Heatmap showing the most relevant microbial genera related to the different types of microbial inoculum. The ARF group was indicated by the blue bar, and the BE group was indicated by the orange bar, and the PE group was indicated by the gray bar.

In accordance with the sPLS-DA analysis **(**Fig. 2**)**, the heatmap showed a clear difference in the rumen microbiota related to the different types of microbial inoculations (Fig. 4**)**, with specific genera strongly related to the ARF inoculum type, differentiating this treatment group from the BE and PE.

### Functional microbial signature by inoculum type

A total of 304 KEGG modules and 202 metabolic pathways were identified using the HUMAnN2. Among these, 201 modules and 170 metabolic pathways showed the relative abundance of > 0.05% across all the samples.

Analysis using microbial protein coding reads indicated that both BE and PE had a significant impact on the abundance of KEGG modules (p < 0.05) related to acyl-CoA synthesis (M00086), type VI secretion (M00334), and methanogenesis (M00347), while PE had a significant impact on KEGGs related to ectoine biosynthesis (M00033) and D-xylose transport (M00215) (Supplementary Table S3).

### Rumen epimural microbiota and its association with host rumen epithelium expression changes

To investigate the relationship between host epithelial expression genes and the epimural microbiota, we performed correlation analysis between gene expression level and microbial taxa abundance using Spearman analysis in R. Microbial taxa were collapsed at the genus or last characterized level and filtered at 0.1% of relative abundance. We found the 30 most significant, unique gene-microbe correlations in the rumen epithelium (p < 0.00001), where the magnitude of correlation (Spearman’s Rho) ranged between 0.994 and 0.999 (Supplementary Table S4).

**Figure 5:**
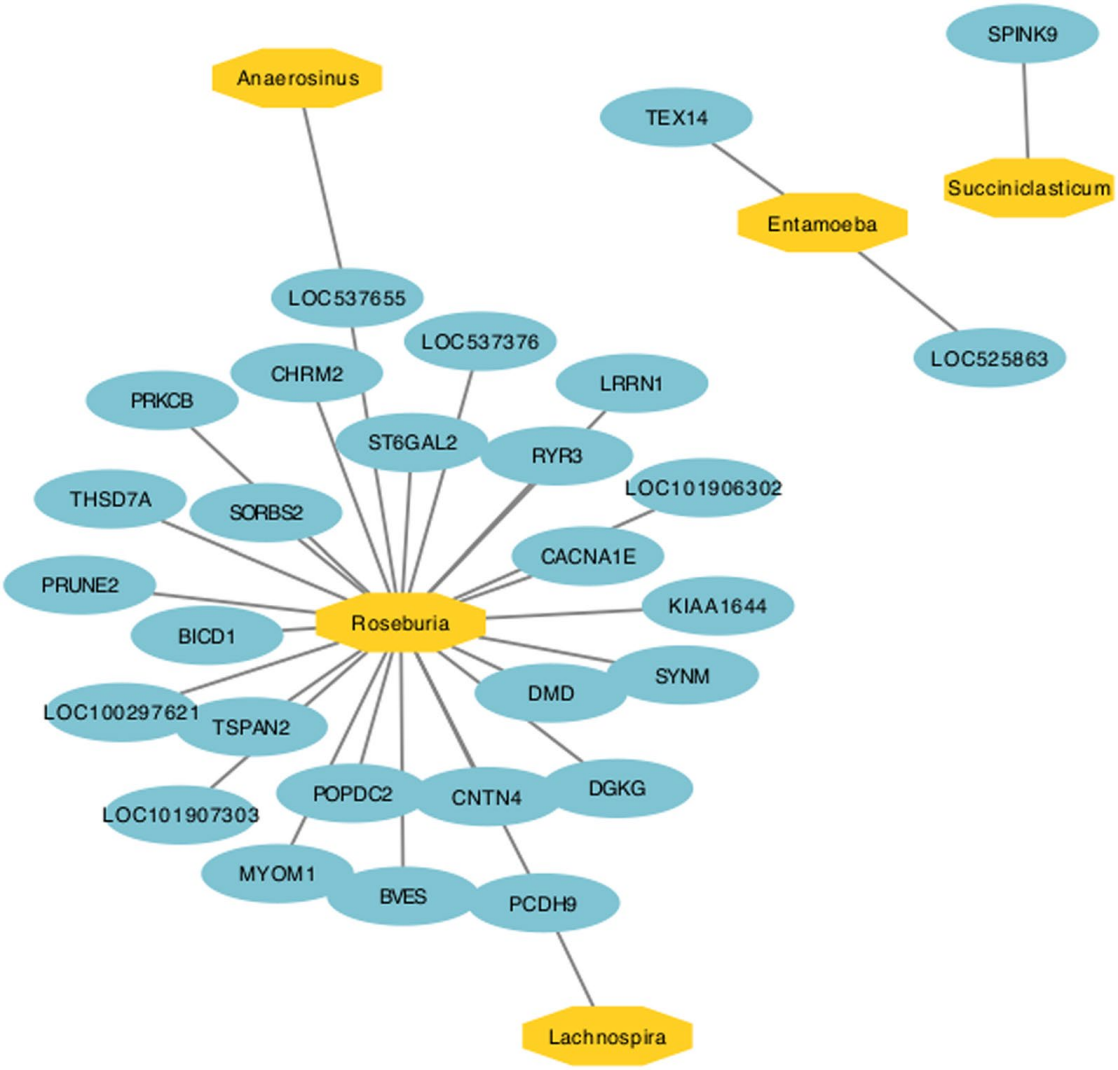
Interactions between host tissue rumen epithelial genes associated with epithelial rumen microbiome. Network visualizing top 30 significant gene-microbe correlations.

The correlations between microbial taxa abundance and host gene expression on the overall network are presented on Fig. 5. Solid edges denote gene-microbe correlations mainly involving the genus *Roseburia* and the genes *CACNA1E* (calcium voltage-gated channel subunit alpha1 E), *THSD7A* (thrombospondin type 1 domain containing 7A), *DMD* (dystrophin)*, PRUNE2* (prune homolog 2 with BCH domain)*, SORBS2* (sorbin and SH3 domain containing 2)*, RYR3* (ryanodine receptor 3)*, BVES* (blood vessel epicardial substance)*, PRKCB* (protein kinase C beta)*, PCDH9* (protocadherin 9)*, ST6GAL2* (ST6 beta-galactoside alpha-2,6-sialyltransferase 2)*, SYNM* (synemin)*, TSPAN2* (tetraspanin 2)*, DGKG* (diacylglycerol kinase gamma)*, CHRM2* (cholinergic receptor muscarinic 2)*, LRRN1* (leucine rich repeat neuronal 1)*, MYOM1* (myomesin 1)*, KIAA1644* (shisa like 1)*, BICD1* (BICD cargo adaptor 1)*, CNTN4* (contactin 4). The genus *Entamoeba* was strongly related to the gene *TEX14* (testis expressed 14, intercellular bridge forming factor). The genus *Succiniclasticum* was related to the gene *SPINK9* (serine peptidase inhibitor Kazal type 9). *Roseburia* and *Lachnospira* were related to the gene *PCDH9* (protocadherin 9) (Supplementary Table S1). We used the GO enrichment analysis for the for DEGs related to microbial taxa. We found no enriched GO terms for *Succiniclasticum* and *Lachnospira*. *Roseburia* was related to two enriched GO terms, both within the category of Cellular Component (GO:0030018 ~ Z disc, 2 genes, p-value < 0.01, and GO:0042383 ~ sarcolemma, 2 genes, p-value < 0.01) (Supplementary Table S1).

## Discussion

This study aimed to elucidate the impact of different types of microbial inoculum on the RE transcriptome and REM in calves. We used an RNA-seq read-based characterization of the rumen epithelium of calves receiving three different types of artificial microbial inoculum. Although rumen microbiome analysis techniques have been mostly based on DNA sequences, RNA-seq analysis can be advantageous by elucidating accurately the active microbial functions^25^.

To our knowledge, this is the first study to use RNA-seq to explore the rumen epimural microbiota and its influence on the rumen epithelium transcriptome in inoculated, pre-weaned calves. Several genes in the rumen epithelia were differentially expressed across the three groups of animals receiving different types of rumen inoculum. Evenness, Shannon’s diversity, and Simpson’s index did not differ across the different microbial inoculum types, while the Observed genera and Chao1 did. The sPLS-DA analysis showed a partial similarity in the microbial taxonomic profile between BE and PE, which differed drastically from the taxonomic profile of the ARF group. Last, we found microbiome-host interactions that indicate the influence rumen microbiome composition on rumen epithelial gene expression.

### Rumen epithelial transcriptome changes in calves treated with different types of microbial inoculum

For the top 5% most highly expressed genes in RE tissue across ARF, BE, and PE groups, 31 of them were uniquely expressed in the ARF group, 20 were uniquely expressed in the BE group, and 6 genes were uniquely expressed in the PE group. These results suggested that different sets of highly expressed genes responded to each inoculum type. The ARF group showed a greater number of uniquely expressed genes than the groups that received the inoculum with different microbial compositions. Compared to ARF, PE had a higher number of DEGs than BE did, suggesting that PE had a higher impact on the rumen epithelium transcriptome expression. PE is a protozoa enriched inoculum, and the functional role of protozoa in the cattle rumen is largely unexplored. Our findings indicated that further investigation on the molecular mechanisms underlying the crosstalk between rumen epithelial and its protozoan population may help yield meaning insights.

Gene ontology (GO) is a widely used and well-documented annotation system that assigns molecular function, cellular component information, and biological process to gene products, allowing the comparison among subcellular structures^26^. In this study, we used the DEGs (Supplementary Table S1**)** for the Gene Ontology analysis in DAVID^27^. We found that the enriched GOs terms between ARF and PE groups were 100% in the category Cellular Component (Bonferroni ≤ 0.05) (Supplementary Table S1). Cellular components refer to the anatomic structures of the cell, where the gene product execute its function, and in our study the most enriched GOs were related to single-organism cellular process (GO:0044763, 62 genes, p-value < 0.01), developmental process (GO:0032502, 47 genes, p-value < 0.01), and anatomical structure development (GO:0048856, 45 genes, p-value < 0.01). Comparatively, animals with increased rumen absorption, as seen in those with high feed efficiency, have an increased expression of genes involved in metabolic processes, tissue morphogenesis, energy-generating pathways^28^; cellular functions^21,28,29^; and in short chain fatty acid (SCFA) absorption and metabolism^30,31^. On the other hand, for the PE group, no significant GO enrichment was detected. Using the unique genes for each group, we did a focused GO terms analysis in the category on biological process (BP) to further explore the effects of the treatments on the rumen epithelial function. We found enriched GO terms in ARF and BE, but not in the PE group. The BE group had several BP enriched terms related to the epithelium (GO:0030855-epithelial cell differentiation, 5 genes, p < 0.007; GO:0002064-epithelial cell development, 3 genes, p < 0.03; GO:0060429-epithelium development, 6 genes, p < 0.008), and related to proteins (GO:0008104-protein localization, 9 genes, p < 0.002; GO:1990778-protein localization to cell periphery, 3 genes, p < 0.05; GO:0072657-protein localization to membrane, 6 genes, p < 0.0002; GO:0019538-protein metabolic process, 11 genes, p < 0.02; GO:1903076-regulation of protein localization to plasma membrane, 3 genes, p < 0.005; GO:0051246-regulation of protein metabolic process, 9 genes, p < 0.003). The ARF group had several BP enriched terms related to the cell localization (GO:0,051,641-cellular localization, 16 genes, p < 0.05; GO:0032879-regulation of localization, 9 genes, p < 0.05; GO:0040012-regulation of locomotion, 6 genes, p < 0.03), and related to mRNA processing (GO:0016071-mRNA metabolic process, 5 genes, p < 0.02; GO:0000398-mRNA splicing, via spliceosome, 3 genes, p < 0.02) (Supplementary Table S1**)**.

### Different inoculum types modified the taxonomic and functional composition of the rumen microbiota

Our results showed that the most abundant epimural microbial genera are prokaryotic. We found a high abundance of the genus *Klebsiella*, that are known for being present in mammary gland of cows and related to mastitis^32^, and has also been detected in samples collected in farms from water, soil, cattle feces, bedding, alleyway, and holding pen^32^. *Klebsiella* is not commonly found in a high abundance on rumen content samples, and the high abundance of this taxa found in our study might be explained by the fact that a different sample type, rumen epithelium, was analyzed in this study. When analyzing the rumen liquid metatranscriptome from the same animals, Park and co-authors^24^ did not find this microbial genus, suggesting its presence is in the rumen epithelium only. Additionally, most of the studies exploring the rumen microbiome use DNA-based 16S rRNA amplicon sequencing methods, while we used whole transcriptome RNA sequencing approach. RNA-sequencing approach has two major advantages over 16S rRNA amplicon sequencing. RNA-sequencing is a random fragmentation based sequencing of the entire transcriptome, which is not limited by any pre-determined genomic regions like the 16S rRNA sequencing. Additionally, RNA-seq has the ability to identify actively transcribed microbial transcripts. For microbial taxa that have low abundance in a community, they might be difficult to identify using DNA-based, 16S rRNA methods. However, if these microbial taxa produce high number of transcripts, they can be identified using RNA-sequencing method. Thus, there can be “microbial blind spots” when different sequencing methods are used. Consistent with this, Park and co-authors^24^ showed that RNA-sequencing and DNA-based 16S rRNA methods showed different microbiota profiles for the same rumen liquid samples, with a sizable number of microbial taxa being identified exclusively by RNA-seq method.

The overall diversity of the RE microbiome was lower in ARF when compared with BE and PE treatments, with a significant decrease in the number of observed genera and in the alpha diversity measured by Chao1. The greater number of observed genera with BE and PE could be due to the live microbial inocula promoting epimural microbial establishment. These results are consistent with the diversity measurements from rumen fluid using a 16S sequencing reported previously for these treatments^33^. However, these differences were not detected in the ruminal fluid from these same calves using RNA-seq^24^, further indicating that the microbial communities in the RE and rumen fluid are different.

The microbial taxa related to the BE treatment have several potential functions. The taxon U29_B03 was found to have a positive correlation with SCFA concentration on the rumen^34^. The UCG−008 is a butyrate producing bacteria belonging to the family Lachnospiraceae^35^. Butyrate is a potent stimulator of epithelial proliferation^36^, and an increase in epithelial proliferation results in an increase in rumen absorptivity and is related to high feed efficiency in cattle^30^. The genus *Synergistes* plays a role in methane production by cooperating with methanogens (*e*.*g*., *Methanomicrobia*), conducting interspecies hydrogen transfer^37^. Since the methane produced in the rumen is not absorbed by the animal, the increase in the methane production represents an energy loss to the animal^19^.

Among the genera related to the PE taxonomic signature, only three genera have previously reported functions in the rumen. *Spirochaeta* plays a role in plant biomass degradation through the secretion of glycoside hydrolases^38^. *Butyrivibrio* encodes a diverse spectrum of degradative carbohydrate-active enzymes (CAZymes), which degrade polysaccharides to yield volatile fatty acids, that are used by the host for growth^39^. Additionally, *Fretibacterium* was found to be positively correlated with greater amounts of long-chain fatty acids, including alpha-linolenic acid, nervonic acid, and palmitic acid, that are related to the growth of specific microbial groups on the rumen^40^.

For the microbial genera related to the ARF taxonomic signature, two genera have known functions. *Alistipes* play a role in the degradation of plant-derived polysaccharides^41^. Members of the genus *Prevotella* have been associated with animals with high feed efficiency and animals with low feed efficiency, indicating that the species within this genus play different roles in rumen fermentation^19^.

According to the sPLS-DA analysis and the alpha-diversity indexes, the taxonomic profile of the RE microbial community is different in calves receiving BE and PE from the animals that received ARF. However, the functional profile shows that the BE and ARF groups are closer to compared to the PE group. The BE and PE groups shared several KEGG modules, and some modules were found exclusively in PE, and no specific KEGG module was found for the ARF group. Studies have shown that the functional profile of the rumen microbiome is more related to the health of the host and the milk production than the taxonomic profile^19,42^. Therefore, despite the perceived composition and functional differences and similarities among the treatment groups, it is still unclear if or how the inoculation of different microbial groups could improve the rumen fermentation and, consequently, improve animal health and production traits.

### Microbiome-host interactions may influence rumen epithelial gene expression

The RE microbiome lies at the interface between the host and its gut environment, and the microbial activity in the RE directly influences the metabolism and physiology of the host animal^43^. The top 30 overall interactions within the rumen epithelial microbiome, which is represented by the abundance of major microbial genera and the DEG, were compared. A total of 38 nodes and 30 significant interactions between the RE microbiome and DEG were significant. The genera *Roseburia*, *Anaerosinus*, and *Succiniclasticum* were considered keystone taxa based on centrality measurements. *Roseburia* was strongly related to the 25 URGs, *Entamoeba* related to 2 URGs, and *Anaerosinus*, *Lachnospira*, and *Succiniclasticum* were related to one URG each (Fig. 5).

The genus *Roseburia* plays a role in starch fermentation, being a prominent butyrate producer^44^, and in sheep was found to have an increased abundance in animals with high feed efficiency^45^. In our study, the enriched GO terms from DEGs correlated to *Roseburia* were related the cell structure (GO:0030018-Z disc) and (GO:0042383-sarcolemma) (Supplementary Table S1), while the DEGs correlated the other microbial genera presented no enriched GO terms. The genus *Anaerosinus* has the ability to ferment glycerol to propionate^46^, and the genus *Succiniclasticum* was reported to convert succinate to propionate^47^. Butyrate and propionate syntheses compete for the same substrate that archaeas use for methanogenesis in rumen, the hydrogen^48,49^. The genus *Entamoeba* comprises protozoan parasites hosted by vertebrates and invertebrates animals^50^. *Entamoeba* spp. have been detected from cattle feces in animals without any clinical symptoms^51,52^. It suggests that these protozoa can occur in cattle in a nonpathogenic form, and that their occurrence might be more common than previously thought. Our study has the first finding of *Entamoeba* spp. on the rumen of cattle. Our study used RNA-based methods to explore the rumen epithelium, and as cited before, it can explain differences in the microbial community composition when comparing with other studies about the theme. However, it is important to highlight that there are no rumen protozoa specific databases, and the bioinformatics analysis match the sequence against organisms described in other environment, which can cause potential misclassification of the microbes.

Our study suggests that different types of microbial inoculum alter the RE transcriptome and metatranscriptome. However short- and long-term implications of these results are not clear. Despite the ARF group having received autoclaved rumen fluid and have lower microbial diversity on RE, more DEGs were found in ARF when compared to PE, but not when compared to BE. Future studies should be conducted to elucidate the mechanisms of dosing on the epimural microbiome and their relation to the RE tissue function. For example, it is still not clear if the KEGG modules found in our study are related to the rumen establishment of the microbial taxa present from the inoculum or whether it is related to the interaction of these microorganisms with the microbes already present in the rumen (*e*.*g*., by predation, competition, etc.). Nonetheless, our findings suggest the possibility of manipulating the rumen epimural microbiome on calves in early life, and that the differences in the epimural microbial community influences the rumen epithelium tissue gene expression. However, these findings pointed out the potential manipulation of the epimural microbiome and RE in calves on early life, with the potential to alter host phenotype in dairy cattle. These results should be confirmed with further long-term studies to evaluate the effects on future productivity and feed efficiency in adulthood.

Our study suggested that different types of microbial inoculum alter the taxonomic and functional composition of the rumen epimural microbiota and transcriptome profile in the RE tissue. However, further studies are needed to better understand the functional impact of exogenous rumen content inoculation in young calves. Specifically, a better understanding is needed for how different types of rumen inoculum could improve rumen fermentation and, consequently, impact the host health, productivity, and efficiency. This work adds empirical evidence suggesting the feasibility of manipulating the RE microbiota through interventions in early life. More rumen developmental studies across different time points using RNA-seq are warranted to better understand how microbial populations and their functions influence host gene expression.

## Conclusions

The rumen of dairy cattle contains microbiota from all domains of life that play a prominent role in digestion and may also affect animal health, production, and efficiency. To evaluate the effects of directed rumen microbial establishment on the gene expression of the rumen tissue and epimural microbiota, we dosed the rumen of pre-weaned dairy bull calves with either bacteria- or protozoa-enriched inoculum from adult cows. This is the first study to show that inoculation with different microbial treatments can alter the expression of genes on the rumen wall and its associated microbiota, which could lead to the development of methods for improving rumen fermentation and host health.

## Methods

### Experimental design and calf management

All animal experimental protocols were conducted in compliance with the ARRIVE (Animal Research: Reporting In Vivo Experiments) guidelines. All animals involved in this study were managed according to the standard practices used at the USDA Dairy Forage Research Center farm throughout the experiment. The animal procedures were approved by The University of Wisconsin’s Institutional Animal Care and Use Committee under protocol A005829. The experimental design, animal procedures, dietary condition, and inoculum preparation were described in our previous studies^23,24^. Briefly, Holstein bull calves (n = 20) at birth were randomly assigned to a 2 × 2 factorial arrangement of treatments over a 4-week period from July to August 2017 and received four different types of rumen microbial inoculum, with five animals per treatment. Samples from the calves provided with the combined inoculum treatment reported were not analyzed in this study. The calves were separated from their dam at birth to keep the animals defaunated, and housed in individual calf hutches with sand bedding at the US Dairy Forage Research Farm in Prairie du Sac, WI.

Calves received 2.5 L pasteurized waste, antibiotic-free milk from day 2 to 7 weeks of age, three times per day, and were offered Vita Plus BSF 18 texturized calf starter (Vita Plus Corp., Madison, WI) for ad libitum consumption from 6 d of age (composition details are reported in Cersosimo and co-authors^23^. From 3 to 6 weeks of age, the calves were orally dosed once weekly with 50 mL of one of the types of treatment inoculum, which was followed by 50 mL 0.7% sterile saline solution, due to veterinarian recommendation. A detailed description of the collection, processing and separation of rumen microorganisms for the inoculum was described in our previous published work^23^. Briefly, rumen contents were collected from four cannulated primiparous cows. Cows were fed once a day with a total mixed ration containing alfalfa and corn silages, ground corn, protein byproduct supplements, and a vitamin/mineral mixture containing monensin. Monensin was included in the starter diet that was fed to calves receiving all treatments at 40 g/ton in order to equalize the monensin intake across treatments. The rumen containing either autoclaved (control; ARF), blended, strained, and centrifuged to create a bacterial-enriched inoculum (BE), or strained and placed in a separatory funnel for 1 h to create a protozoal-enriched inoculum (PE).

### Sample collection

The calves were fasted overnight and euthanized at 9 weeks of age at the University of Wisconsin Meat Science Laboratory by a penetrating captive bolt followed by exsanguination. After animal euthanasia, zip ties were used to isolate the stomach compartments. Four randomly selected calves from each treatment were subjected to rumen epithelial tissue collection for host transcriptome and microbial metatranscriptome analyses. The rumen epithelium from the caudal ventral region of the rumen was collected immediately after euthanasia and rinsed in PBS to remove the remaining feed particles. Rumen epithelial samples were cut with sterilized scalpels into 4–5 mm^2^ fragments, put into Eppendorf safe-lock tubes (Eppendorf North America, Hauppauge, NY), snap-frozen in liquid nitrogen and stored at −80 °C for RNA sequencing analysis.

### RNA extraction, quantification, and whole transcriptome sequencing from the rumen epithelium tissue

The RE tissues were ground into a fine powder in liquid nitrogen using a mortar and pestle. Total RNA was extracted from the tissue homogenate following the miRNeasy protocol with a QIAcube instrument (Qiagen US). The quality of the extracted RNA was checked using Bioanalyzer RNA 6000 Nano Kit on the Agilent 2100 Bioanalyzer (Agilent Technologies, US), and samples with RIN ≥ 8 were pursued for RNA quantification using Qubit 3.0 Fluorometer (Thermo Fisher, US). Library preparation was done using Illumina TruSeq Ribo-zero Gold Kit following the manufacturer’s instructions after removal of ribosomal RNAs from the host cattle sample. One µg of total RNA from each sample was used for library preparation, quantification of the prepared libraries was performed using a Kapa Quantification Kit (Kapa Systems) in an QuantStudio5 RT-qPCR instrument (ThermoFisher, US), and the libraries were further normalized to ensure equal quantity before sequencing. Paired-end reads (2 × 75 bp) were obtained using an Illumina NextSeq 500 instrument with 150-cycle high-output kit.

### Reverse transcriptase qPCR (RT-qPCR) verification of RNA sequencing results

Five randomly selected differentially expressed genes (DEGs) identified by RNAseq were analyzed by RT-qPCR analysis. Two of these (*LY6G6E* and *PCDH7*) were selected from the comparison of Control x BE, and the rest of the three (*CA3, CFL2 and GPX3*) were selected from the comparison of Control x PE. *LY6G6E* is one of the leukocyte antigen-6 (LY6) genes as part of the major histocompatibility complex class III region on chromosome 6^53^. *PCDH7* encodes a protein with an extracellular domain, which is thought to be an integral membrane protein functioning in cell-cell recognition and adhesion^54^. *CA3* belongs to a gene family encoding proteins that catalyze the hydration of CO_2_ to generate protons and bicarbonate ions for cellular ion transport and pH homeostasis^55^. CFL2 encodes a protein that controls actin polymerization and depolymerization in a pH-sensitive manner^56^. GPX3 encodes a secretory enzyme that plays role in protecting the cells against oxidative stress^57^. cDNA synthesis was performed using 2 μg of RNA with High-Capacity cDNA master mix (ThermoFisher Scientific, US). Gene-specific, Taqman assay probes were ordered from ThermoFisher (ThermoFisher Scientific, US). All PCR reactions were performed using the QuantStudio5 (ThermoFisher Scientific, US). The thermal cycler steps are as follows: one step of UNG treatment at 50 °C for 2 min, followed by an initial denaturation/activation step at 95 °C for 2 min, then 40 cycles at 95 °C for 15 s and 60 °C for 60 s. The analyses were carried out in triplicate for each data point. The fold change in gene expression was obtained following normalization to two reference genes, *ACTB* and *HMBS*. These two reference genes were found to be very consistent in cattle^58^. The relative quantification of gene expression was determined using the 2^−ΔΔCt^ method^59^.

### Mapping of RNA sequencing raw reads and accessing differential gene expression analysis

FastQC was used to check the quality of the raw reads (https://www.bioinformatics.babraham.ac.uk/projects/fastqc/), and the raw reads were filtered to remove those shorter than 50 bp. For sequence alignment, the genome reference and the annotation file of *Bos taurus* were used (NCBI, ARS-UCD v.1.2)^60^ and raw sequencing reads were aligned using STAR^61^. Cufflinks^62^ was used to determine the expression level of mRNAs in each sample and to calculate the Fragments Per Kilobase of transcript per Million mapped reads (FPKM) for each gene. The total number of expressed genes was calculated using a FPKM cutoff value of 1. Differential expressed gene (DEG) analysis across the groups (ARF, BE, and PE) was performed using cuffdiff module in cufflinks v. 2.2^62^. DEGs analysis was done for the comparison between control group (ARF) and the groups BE and PE, and between BE and PE. Gene function annotation and pathway analysis were performed using DAVID^27^ and stringDB v.11.5^63^. The most highly expressed genes (top 5%) were first identified for each sample using FPKM values. Then, the most abundantly expressed genes across the three groups and the most highly expressed genes that are unique to each group were identified. To gain new insights into the underlying biological functions of DEGs, the Gene ontology (GO) pathway analysis was performed using stringDB v.11.5^63,64^ and used to analyze the identified DEGs (p ≤ 0.05).

### Data analysis of microbial community

The cattle unmapped, paired-end reads were used for microbial community analysis. Microbial taxa classification was done by Kraken2^65^ through custom-built reference using the SILVA138.1 dataset (https://www.arb-silva.de/documentation/release-1381/). Genus level classification was followed for downstream analysis. To enrich the reads from microbial coding transcripts, the cattle unmapped reads were mapped to the reference database provided by SortMeRNA (version 2.1b)^66^. The unmapped reads after this step were considered as microbial protein coding reads.

### Functional annotation of the microbial coding reads

The functional profiling of metatranscriptomic reads was performed using HUMAnN2 (http://huttenhower.org/humann2). HUMAnN2 provides a curated profiling for presence, absence, and abundance of microbial pathways (www.kegg.jp/kegg/kegg1.html) Refs.^67–69^ in a community, and it allows the description of the metabolic activity of a microbial community^70,71^.

### Statistical analysis

To investigate alpha-diversity in the metatranscriptomic data, Shannon’s diversity, Simpson's index, and Evenness were calculated, including richness indices (number of observed genera and Chao1 richness estimates) using the rarefied read count table of rumen epithelial microbiota at the genus level. The mixMC multivariate method implemented in the mixOmics R package v. 6.22.0 was used to identify associations between microbial taxonomic profiles and the treatments. For this analysis, we considered only microbial taxa with relative abundance > 0.01% across all the samples. Then, we used sparse partial least square discriminant analysis (sPLS-DA)^72^ to identify taxonomic microbial signatures related to each treatment group (ARF, BE, and PE) with 95% of confidence.

By comparing the significant interactions between the rumen epimural microbiota and the RE gene expression, exclusive features and interactions were selected using the R package, Co-expression Differential Network Analysis (CoDiNA) v. 1.1.2^73^. The top 20 significant interactions (p < 0.05) were visualized using Cytoscape v.3.9.1^74^. We used stringDB^63^ to perform the Gene Ontology (GO) analysis for the DEGs correlated to bacterial taxa and the uniquely highly expressed genes in RE with different inoculum treatments. The redundant GO terms were summarized using REVIGO^75^. We used DAVID^27^ to perform the biological process (BP) analysis, since StringDB does not allow to perform a GO analysis focused in specific GO domains.

### Ethics approval

The animal study was reviewed and approved by the IACUC committee of University of Wisconsin-Madison under protocol number A005829. Aside from the inocula treatments included in this study, all the animals were raised according to the standard practice adopted by the US Dairy Forage Research Center, USDA.

### Supplementary Information

Supplementary Legends.

Supplementary Table S1.

Supplementary Table S2.

Supplementary Table S3.

Supplementary Table S4.

## Data Availability

The original contributions presented in this study are include in the article. RNA sequencing raw reads were deposited at NCBI SRA with the accession number of PRJNA995806 (https://dataview.ncbi.nlm.nih.gov/object/PRJNA995806?reviewer=bg70svivlh38bhuuk3l96cmb84). Further inquiries can be directed to the corresponding authors.
