# Supplementary material for: Microbial inoculum effects on the rumen epithelial transcriptome and rumen epimural metatranscriptome in calves
Source: Sci Rep. 2024 Jul 23;14:16914. doi: 10.1038/s41598-024-65685-y (PMC11266570; doi:10.1038/s41598-024-65685-y)
Supplement: Supplementary file 1 — Supplementary Legends. [file 41598_2024_65685_MOESM1_ESM.docx]

**Microbial inoculum effects on the rumen epithelial transcriptome and rumen epimural metatranscriptome in calves**

P. Fregulia, ^1,2^, T. Park ^1,2,3^, L.M. Cersosimo ^1,2,4^, G. I. Zanton ^1^, W. Li ^1*^

^1^*United States Department of Agriculture (USDA) - Agricultural Research Service, Dairy Forage Research Center, Madison, WI, United States*

^2^*Oak Ridge Institute for Science and Education, Oak Ridge, TN*

*^3^Present address: Tansol Park, Department of Animal Science and Technology, Chung-Ang University, Anseong, South Korea*

*^4^Present address: Laura Cersosimo, Brigham and Women’s Hospital, Boston, MA, USA*

* Corresponding author: Wenli Li wenli.li@usda.gov

**Supplementary table S1.** Gene Ontology (GO) for the differentially expressed genes, and for the genes related to microbes in the Network analysis.

**Supplementary Table S2.** List of all the taxa identified in the metatranscriptomic analysis of the rumen epithelium, at phylum and genus level.

**Supplementary table S3.** List of KEGG modules identified in the ARF, BE and PE groups.

**Supplementary Table S4.** The most significant and unique gene-microbiome correlations in the rumen epithelium
